# A relationship that makes life worth-living: levels of value orientation explain differences in meaning and life satisfaction

**DOI:** 10.1016/j.heliyon.2022.e08802

**Published:** 2022-01-24

**Authors:** Anastasia Besika, Jonathan W. Schooler, Bas Verplanken, Alissa J. Mrazek, Elliott D. Ihm

**Affiliations:** a Developmental Psychology, University of Zurich, Zurich, Switzerland; b Psychological and Brain Sciences, University of California, Santa Barbara, USA; c Department of Psychology, University of Bath, Bath, United Kingdom; d Department of Psychology, University of Texas at Austin, USA

**Keywords:** Latent value profiles, Meaning in life, Sources of meaning, Latent profile analysis, Satisfaction with life, Well-being

## Abstract

When people talk about their values they refer to what is meaningful to them. Although meaning is associated with life satisfaction, previous studies report inconsistent results regarding the association of values and well-being. A cross-sectional study (*N* = 276) addresses the research question, do values influence experiences of meaning and subjective evaluations of life satisfaction? To assess whether providing a definition of “meaningful” is necessary when employing meaning measures, we assigned participants to condition where some provided their definition and others read a definition of “meaningful”. All participants described a recent meaningful experience; they characterized it with sources of meaning; they read descriptions of 10 values and assessed the degree those were relevant to their experience; and they completed meaning and life satisfaction measures. Findings, which were unaffected by reading a definition of “meaningful”, indicated that the most common source of meaning (Family) was associated positively with the value of Tradition and negatively with the value of Universalism. Latent Profile Analysis identified three profiles denoting participants’ level of value orientation, which explained interindividual differences in average levels of meaning and life satisfaction variables. Participants who associated their meaningful experience with the 10 universal values at a high level scored higher in the meaning and life satisfaction measures than those who associated their experience to the 10 universal values at a low level. The present work advances knowledge regarding the relationship between meaning, values and life satisfaction and validates previous studies reporting on meaning as a marker of well-being.

## 1 Introduction

Life may come with extraordinary meaningful events as well as meaningful moments weaved into daily routines (Heintzelman and King, 2019). What characterizes meaningful experiences that make life seem worth-living?

People experience meaning on the affective, cognitive and motivational levels of functioning (e.g., O'Connor and Chamberlain, 1996). On the affective level, research evidence indicates that meaning emerges from positive affect (King et al., 2006; King and Hicks, 2021) and can transform negative into positive affect (Wong, 2012). On the cognitive level, studies show that meaning acts as an environmental decoder and helps people with processing information, coordinating their action and communicating complex concepts (Heintzelman and King, 2014). On the motivational level, although there are suggestions that people's values may capture what is meaningful to them (Reker, 2000; Reker and Wong, 1988), empirical evidence show no association between the 10 universal value domains (Schwartz, 1992) and measures of meaning (Steger et al., 2006).

In the present article, we investigate the relationship between values and well-being and provide primary empirical evidence that suggests a strong association between the degree values, as a dynamic pattern, influence average levels of meaning and life satisfaction. We assess value patterns using Schwartz's (1992) 10 value domains model. Toward the validation of our study, and to address expressed skepticism over previous studies that used meaning measures (Park, 2010; Leontiev, 2013), we assess whether relying on participants' subjective interpretations of “meaningful” undermines results.

### 1.1 Meaning in life and values

According to recent theoretical developments*, meaning in life* or *meaning* comprises *comprehension, mattering* and *purpose*: Comprehension refers to having clarity about one's life events; Mattering refers to having a sense that one's life is significant; Purpose refers to pursuing goals that are in line with one's values (George and Park, 2017; Martela and Steger, 2016). In this way, values may be considered the motivational aspect of meaning (Reker and Wong, 1988). Values refer to mental representations of ideal states that may motivate and guide behavior (Maio, 2010; Schwartz, 1992). Over the last 30 years, cross-cultural studies have been showing that people share 10 value domains including Stimulation, Self-direction, Achievement, Hedonism, Power, Security, Conformity, Tradition, Benevolence and Universalism. Each value domain represents a number of single values and together they form a circular structure. Conservation, self-enhancement, openness to change and self-transcendence are four motivational orientations that underlie the circular structure, which represents 57 single values in total (Schwartz, 1992).

### 1.2 Dimensions of meaning

There is a conceptual overlap between the four underlying motivational orientations of values (Schwartz, 1992) and an early conceptualization that suggests that meaning has a depth dimension. Depth of meaning refers to four levels, each corresponding to a different type of motivation and set of values (Reker and Wong, 1988). Both theories suggest that the type of experiences people find meaningful correspond to certain values (Table 1). Another conceptual dimension of meaning in life is breadth, which refers to the number of areas people seek meaning or the number of *sources of meaning* (O'Connor and Chamberlain, 1996). Studies that assess the relationship between sources of meaning and well-being and explore the elements that add meaning to daily experiences (e.g. Ebersole and DeVogler-Ebersole, 1986) suggest that people are more satisfied when they have diversity in the areas that provide them with meaning, compared to deriving meaning from a single source. Is there an association between breadth and depth of meaning? Do the two meaning dimensions influence the experience of meaning and life satisfaction? How does the structure of values influence well-being?

**Table 1:** Values associated with depth of meaning and the motivational orientation that underlies Schwartz's 10 basic value domains.

Value domains	Depth of meaning (Reker and Wong, 1988)	Motivational orientation (Schwartz, 1992)
Security, Conformity, Tradition	*Level 1:* Comfort	Conservation
Achievement, Hedonism, Power	*Level 2:* Enhancement of personal potential	Self-enhancement
Stimulation, Self-direction	*Level 3:* Serving others in the immediate environment	Openness to change
Benevolence and Universalism	*Level 4:* Serving the universal good	Self-transcendence

### 1.3 Value pattern

A person's value pattern represents what is important to them and influences their daily routines (Besika et al., 2021; Verplanken and Sui, 2019). Studies suggest that from early childhood, people gradually integrate the circular structure of the 10 universal values and assign different priorities to each one at different stages of their development. For example, a longitudinal study with children between 7 and 11 year of age shows that the value of Security is predominantly important to young children as they need to feel safe in the world, whereas the value of Self-direction develops as they become familiar with their environment and want to explore it further (Cieciuch et al., 2016). Another series of longitudinal studies with adolescents and adults that investigated intraindividual change in value priorities across time, indicate that value priorities change systematically after impactful life events. For example, when values of Benevolence decreased in importance values of Universalism increased in importance (Bardi et al., 2009), even though these values are underlain by the same motivational orientation. Overall, conservation and self-transcendence values increase in importance and openness to change and self-enhancement values decrease in importance across the life span (Ritter and Freund, 2014; Schwartz 2006).

In spite of the conceptualization of values as a dynamic system and empirical evidence suggesting that values fluctuate systematically, researchers typically use measures that involve pairwise comparisons of values and ask participants to rate the degree to which one value is more important to them in comparison to others (e.g., Oishi et al., 1999; Sortheix and Schwartz, 2017). Consequent analysis when assessing their relationship to well-being focuses on participants' value priorities and dismisses the role of less important values at the time of assessment. Assuming that value priorities form a cognitive pattern (Schwartz, 1992), that fluctuates systematically (Bardi et al., 2009), single value priorities do not provide the whole picture of the influence a person's value pattern has on their well-being (Besika et al., 2021). These methodological limitations may explain the results in a study that assessed the relationship between the 10 value domains and the two subscales of the Meaning in Life Questionnaire (MLQ) of *presence* and *search* for meaning (Steger et al., 2006) and found no correlation, which contradicts findings indicating systematic associations between values and important life events (Bardi et al., 2009). Could another methodology detect the suggested association between values and meaning? Moreover, previous studies that focus on value priorities report a negative association between certain values and life satisfaction (e.g., Kasser and Ahuvia, 2002; Sortheix and Schwartz, 2017). Do people's latent value patterns explain variance in life satisfaction better than their value priorities?

### 1.4 The present study

In the present study, we addressed the research question; do values influence the experience of meaning and life satisfaction? Firstly, we investigated whether providing a definition of “meaningful” influenced participants' responses to our meaning measures, as previous studies that assess meaning received skepticism that concerns participants' subjective interpretation of measures. For example, the item in the Meaning in Life Questionnaire “I am searching for meaning in my life” (Steger et al., 2006) could potentially generate a variety of responses that depend on subjective interpretations of what is meant by “searching for meaning”. Secondly, we aimed to test three hypotheses. The first hypothesis is based on the theoretical assumptions that 10 value domains influence people's goals and behavior globally to a different degree (Schwartz, 1992) and that when certain values are highly important to a person others are less important (Bardi et al., 2009). *Hypothesis 1:* The most common source of meaning is positively associated with at least one of the 10 value domains and is negatively associated with at least another one of the 10 value domains. The second hypothesis assumes that people integrate the 10 value domains into their self-concept as a pattern that denotes their value priorities and influences their goals and daily actions (Besika et al., 2021; Verplanken and Sui, 2019). *Hypothesis 2:* People are distinguished by their latent value patterns. The third hypothesis assumes that people's values underlie their meaningful experiences (Reker and Wong, 1988) and that meaning is positively associated with life satisfaction (King et al., 2006). *Hypothesis 3:* People's latent value profiles explain variance in meaning and life satisfaction measurements. In addition, we explored the questions: a) is there a relationship between breadth of meaning (i.e., the number of sources of meaning) and depth of meaning (i.e., the four motivational orientation of values)? and b) is there an association between the two meaning dimensions and measures of meaning and life satisfaction?

#### 1.4.1 Addressing methodological challenges

Research evidence shows that values are not typically salient in people's awareness (Verplanken and Holland, 2002). Thus, to investigate values we employed strategies to address the limitations imposed by their latent nature. In our study, we aimed to activate participants' values by engaging them in describing a recent meaningful experience and in evaluating the degree this experience contributed to the fulfillment of the 10 universal values. Another challenge was to eliminate researcher's biases from the process of compiling a list of sources of meaning (Barbour, 2001) in order to investigate breadth of meaning. In previous studies, researchers typically either used a reductive or a deductive approach (Fereday and Muir-Cochrane, 2006). In the reductive approach the list of sources of meaning was data-driven, where researchers coded participants' essays on what they considered meaningful (e.g., Lambert et al., 2013). The reductive approach involved providing participants with a theory-driven list of sources of meaning (e.g., Reker and Woo, 2011). To prevent imposing personal perceptions on our measure, we compiled a comprehensive list of sources of meaning from 15 previous studies, including an unpublished study conducted by university students. The outcome was a list of semantically unique items that allowed participants to code directly their accounts of experiencing meaning, without the need to transcribe qualitative data.

## 2 Materials and methods

### 2.1 Participants and procedures

The study was reviewed by the University's Institutional Review Board of Human Subjects Committee and was exempt from an ethical assessment, as it did not involve any risk to humans. Procedures were performed in accordance with the ethical standards as set forth in the 1964 Declaration of Helsinki and its later amendments. We determined the sample size based on a priori power calculation in G∗Power (Faul et al., 2007) for means difference between two independent groups. The analysis showed that we could expect to observe a medium effect size of *d* = .50 (Boer, 2017) with *alpha* = .05 and 95% power, with a minimum sample of *N* = 210. A total of 296 volunteers entered the Amazon's Mechanical Turk survey in exchange of $2. Eight participants did not consent to the study and 12 responses did not relate to the request to describe meaningful moments. The final sample was *N* = 276 (age *M* = 36.83, *SD* = 11.16, females = 137).

Participants gave their informed consent to participate in the study and were randomly assigned to condition. In group 1 (read definition) (*n* = 146) participants read the following definition of “a meaningful experience”, based on a recent definition of meaning in life (George and Park, 2017):

*“A meaningful experience adds comprehension, mattering and purpose to one's life. (a) When we experience comprehension, we feel that life makes sense, things seem clear and everything is as it should be. In contrast, when we experience low comprehension, life seems incoherent and unclear. (b) When we experience mattering, we feel that our actions are in line with our entire life. Individuals with a low mattering feel that their existence makes no difference in the world. (c) When we experience purpose we engage with life and have clarity over what we strive for. Individuals experiencing low purpose feel that nothing seems worthwhile.”*

In group 2 (wrote definition) (*n* = 130) participants provided their own definition of “a meaningful experience”. Participants in both groups continued by describing their most meaningful experience of the “last two weeks”. Next, participants rated how meaningful their experience was and characterized it with an unrestricted number of sources of meaning from a 35-item list. Then, participants read descriptions of the 10 value domains and rated the degree those were relevant to their experience. Finally, participants completed three well-being scales and answered demographic questions.

### 2.2 Measures

#### 2.2.1 Value domains

Using the single values that represent each value domain (Schwartz, 1992) we created 10 brief descriptions (e.g., “Hedonism: a sensuous gratification associated with a sense of *feel-good, fun, happiness, indulgence, leisure* and *pleasure*”) (see Appendix A: Tables A4 - A5). Participants rated the degree each of the 10 value domains was associated with their meaningful experience, on a 7-point Likert scale, ranging from 1 *(not at all)* to 7 (*very much*). The estimated reliability of the 10 value items was high (Cronbach's *alpha* = .90).

#### 2.2.2 Breadth of meaning

We reviewed the literature and compiled a list of sources of meaning from studies that investigated breadth of meaning. This process resulted in a total of 106 items (Appendix A: Tables A1 - A3). We condensed the list in two steps. Firstly, we excluded 52 items, which we observed were identical to the single values described by Schwartz (1992) (e.g., fun, happiness, indulgence, leisure and pleasure). Secondly, we merged the items that were semantically unique (e.g., *work* and *occupation* = “work”). Finally, we gave brief descriptions to the remaining 35 sources (e.g., “*Flow*: losing sense of time while doing something smoothly and effortlessly”). Participants coded their experiences with an unrestricted number of sources of meaning using 1 and 0. A numeric variable was computed by adding the number of sources of meaning participants used to characterize their meaningful experience.

#### 2.2.3 Depth of meaning

Depth of meaning helped to explore the relationship between the motivational orientation of values and the well-being variables. A four level categorical variable was dummy-coded based on the values’ motivational orientation. Values corresponding to conservation (Security, Conformity, Tradition) were coded with 1; to self-enhancement (Achievement, Hedonism, Power) with 2; to openness to change (Stimulation, Self-direction) with 3; and to self-transcendence (Benevolence and Universalism) with 4 (Schwartz, 1992; Schwartz and Bilsky, 1987).

#### 2.2.4 Meaningfulness

Participants rated how meaningful their experience was on a 7-point Likert scale, ranging from 1 *(not at all)* to 7 (*very much*).

#### 2.2.5 Well-being

The *Multidimensional Existential Meaning Scale* (MEMS; George and Park, 2017) was used to measure meaning in life as comprising comprehension (Cronbach's *alpha* = 0.94), mattering (Cronbach's *alpha* = 0.85) and purpose (Cronbach's *alpha* = 0.89). Participants rated 15 items (e.g., “I have overarching goals that guide me in my life”). The MLQ (Steger et al., 2006) was used to measure the dimensions of presence (Cronbach's *alpha* = 0.91) and search for meaning (Cronbach's *alpha* = 0.94). Participants rated 10 items (e.g., “I understand my life's meaning”). The *Satisfaction with Life Scale* (SWLS; Diener et al., 1985) (Cronbach's *alpha* = .93) has been widely used in studies as a well-being measure. It measures life satisfaction as a subjective evaluation of one's life as a whole. Participants rated five items (e.g., “In most ways my life is close to my ideal”). All well-being measures were rated on a 7-point Likert scale, from 1 *(very strongly disagree)* to 7 (*very strongly agree*).

### 2.3 Analytic strategy

Analyses was conducted in SPSS Statistics (version 23.0.0) and RStudio (version 1.4.1106) (R Core Team, 2020). To determine whether analyses would require group comparison, we first compared means of well-being measures across the two conditions to test whether reading a definition of “meaningful” had an effect. Correlation analysis helped us explore our questions regarding the relationship between breadth and depth of meaning and well-being. In contrast to the typical value-priority-centered approach, we followed a person-centered approach toward testing our three hypotheses. Once we identified the most common source of meaning people associated with their meaningful experience, we conducted multiple logistic regressions to test whether this source was significantly associated with any of the 10 value domains *(Hypothesis 1).* Latent Profile Analysis (LPA) revealed unobserved subgroups based on participants’ ratings of the 10 value domains, in relation to their meaningful experience *(Hypothesis 2)*. In aiming to eliminate research biases from our investigation we relied on a mathematical evaluation to determine the number of latent value profiles (LVPs) that best represented the data. Finally, we used analysis of variance and multiple group comparisons to test whether LVPs explained variance in meaning and life satisfaction measures (*Hypothesis 3*).

## 3 Results

### 3.1 Definition effect

Shapiro-Wilk tests revealed that the data was not normally distributed as the *W* values were all significant for the well-being scores. Thus, we conducted nonparametric *Mann-Whitney U tests* (Mann and Whitney, 1947; Wilcoxon, 1945) to compare the means of MLQ, MEMS subscales and the SWLS in the two conditions. Table 2 reports the results in detail that showed no significant differences across conditions. Given that reading a definition of “meaningful” had no effect, we continued by utilizing the whole dataset.

**Table 2:** Mann-Whitney U test for two independent samples explored differences between conditions.

Measure	Condition	Mean	SD	U	p
MLQ_Presence	1	5.2	1.40	9280	.751
	2	5.2	1.40		
MLQ_Search	1	4.8	2.40	9166	.625
	2	4.8	2.15		
MEMS_Comprehension	1	5.2	1.60	9470	.976
	2	5.4	1.55		
MEMS_Purpose	1	5.6	1.15	9730	.716
	2	5.6	1.20		
MEMS_Mattering	1	4.8	1.40	9112	.568
	2	4.8	1.60		
SWLS	1	4.2	1.80	9711	.738
	2	5.0	2.00		

*Note.* MLQ = Meaning in Life Questionnaire; MEMS = Multidimensional Existential Meaning Scale; SWLS = Satisfaction With Life Scale; Condition 1 = group that read a definition = 130; Condition 2 = group that provided a definition = 146; SD = standard deviation; U = Mann-Whitney U; *p* = *p*-value.

### 3.2 Breadth of meaning and well-being

Spearman's rank-order correlations were used to measure the strength of association between the two dimensions of meaning, breadth and depth, and the relationship of breadth with the three well-being measures. There was no association between breadth and depth of meaning (*r* = 0, *p* = .998). However, breadth demonstrated small significant correlations with MLQ, MEMS and SWLS, with the exception of the presence of meaning subscale of the MLQ (*r* = .01, *p* = .27). Search for meaning: *r* = .14, *p* < .001; Comprehension: *r* = .12, *p* < .001; Purpose: *r* = .13, *p* < .001; Mattering: *r* = .05, *p* < .001; and the SWLS, *r* = .08, *p* < .001.

### 3.3 Depth of meaning and well-being

We correlated the means of the 10 value domains with the means of the MLQ and MEMS subscales as well as with the means of the SWLS and of ratings of *how meaningful* the experience was (Table 3). Most of the 10 value displayed small significant positive correlations with all the measures. There were fewer significant correlations with the presence of meaning (MLQ) and the purpose (MEMS) subscales. All 10 value domains correlated significantly with the meaningfulness of the experience. Furthermore, we performed analysis of variance to explore whether the well-being scores varied across depth of meaning or the four motivational orientations of the 10 value domains. Significant *F* values in Levene's tests indicated that the homogeneity of variance assumption was violated. Hence, instead of performing ANOVA we conducted the nonparametric Kruskal–Wallis test (Kruskal and Wallis, 1952). Non-significant results indicated that there were no distinct associations between the four underlying motivational orientations of values and the well-being measures in our sample (Appendix B: Table 1).

**Table 3:** Spearman's correlations between the 10 value domains, meaning and life satisfaction measures and “How meaningful” ratings.

Value Domain	MLQ		MEMS			SWLS	Meaningful
	Presence	Search	Comprehension	Purpose	Mattering		
Self-direction	.12∗	.19∗∗	.27∗∗	.21∗∗	.26∗∗	.28∗∗	.23∗∗
Stimulation	.11	.27∗∗	.23∗∗	.24∗∗	.28∗∗	.23∗∗	.19∗∗
Hedonism	.11	.19∗	.27∗∗	.20∗∗	.25∗∗	.23∗∗	.24∗∗
Achievement	.08	.18∗	.21∗∗	.14	.22∗∗	.23∗∗	.20∗∗
Power	.06	.27∗∗	.21∗∗	.11	.25∗∗	.27∗∗	.15
Security	.20∗∗	.20∗∗	.37∗∗	.27∗∗	.29∗∗	.34∗∗	.34∗∗
Conformity	.06	.21∗∗	.25∗∗	.12	.24∗∗	.24∗∗	.14
Tradition	.09	.29∗∗	.28∗∗	.13	.22∗∗	.21∗∗	.24∗∗
Benevolence	.19∗∗	.16	.39∗∗	.20∗∗	.31∗∗	.28∗∗	.33∗∗
Universalism	.13∗	.30∗∗	.26∗∗	.28∗∗	.28∗∗	.24∗∗	.26∗∗

*Note: N = 276;* MLQ = Meaning in Life Questionnaire; MEMS = Multidimensional Existential Meaning Scale; SWLS = Satisfaction With Life Scale; Meaningful = ratings relating to how meaningful participants' experience was; *∗p < .05, ∗∗p < .001* after Bonferroni alpha correction.

### 3.4 Sources of meaning

**Figure 1:**
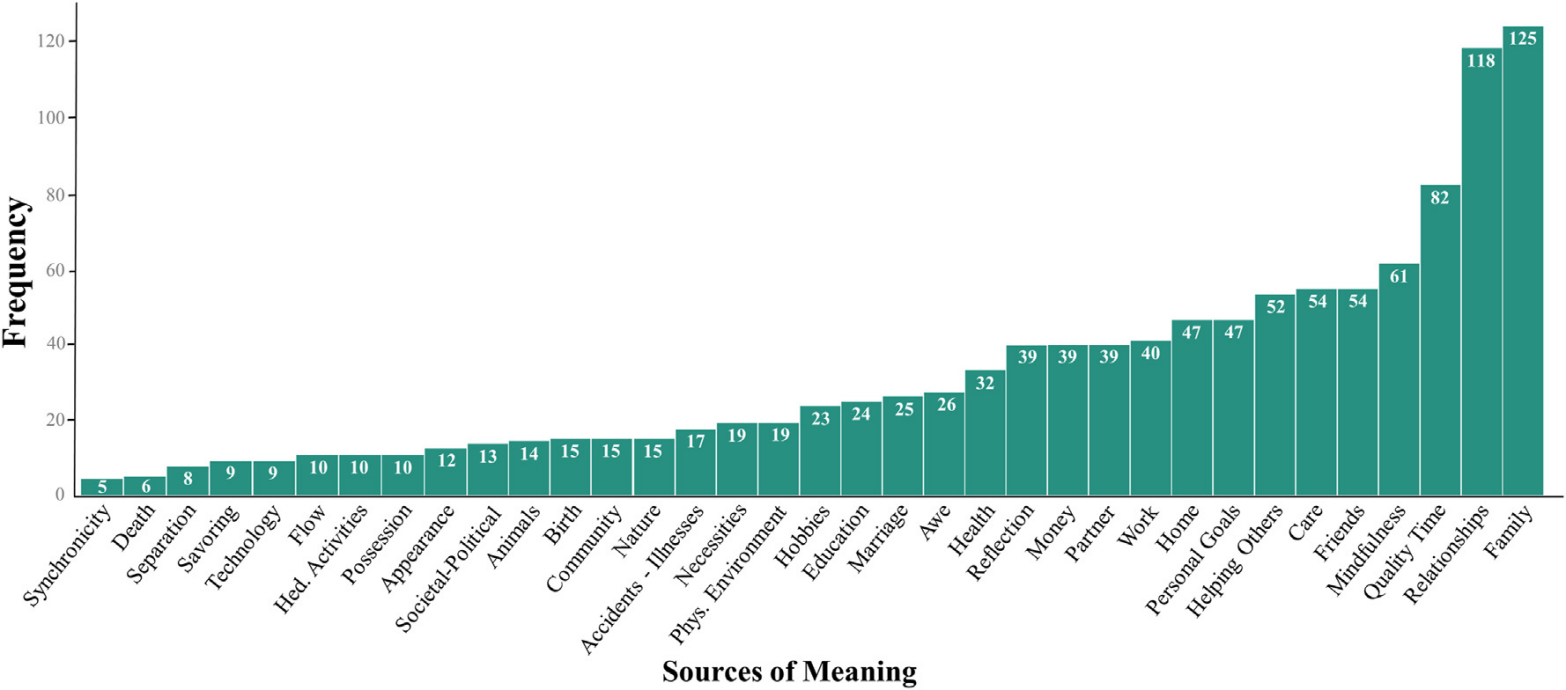
Frequencies of 35 sources of meaning associated with meaningful experiences.

We calculated the frequencies of the sources of meaning that participants used to characterize their most meaningful experience with (Figure 1). All 35 sources of meaning were included in the frequency pattern. “Family” (i.e., people you are related to or feel close to, like parents or children whether you live together or not) was the most common source.

### 3.5 Hypothesis 1: sources of meaning may correlate positively with some and negatively with other values

We conducted binary logistic regression analysis where the most common source of meaning (“Family”) was regressed on the 10 values as multiple predictors. A significant Likelihood ratio and a non-significant *Hosmer & Lemeshow* suggested that the model was a good fit to the data. Table 4 presents results confirming our *hypothesis*, as the value of Tradition was positively associated with “Family”, whereas the value of Universalism reduced the likelihood of “Family” being a source of meaning. Every unit of increase in Tradition increased the odds of “Family” characterizing meaningful experiences by 34%; every unit of increase in rating Universalism reduced these odds by 28% (Peng et al., 2002). As expected, the most common source of meaning amongst participants was associated positively with a value domain and negatively associated with another value domain.

**Table 4:** Results of logistic regression analysis with the 10 universal value domains as the predictors of “Family”, the most frequent source of meaning.

Predictor	β	*S.E.*	Wald	*df*	*p*	Exp(B)
Self-Direction	-.07	.11	.35	1	.56	0.94
Stimulation	.06	.12	.30	1	.59	1.07
Hedonism	.00	.10	.00	1	.97	1.00
Achievement	-.25	.11	5.61	1	.02	0.78
Power	-.17	.13	1.71	1	.19	0.85
Security	.02	.10	.04	1	.85	1.02
Conformity	.12	.13	.86	1	.35	1.13
Tradition	**.51**	.12	18.80	1	**.00∗**	1.66
Benevolence	.19	.10	3.84	1	.05	1.21
Universalism	**-.33**	.11	8.44	1	**.00∗**	0.72
Constant	.01	.42	.00	1	.98	1.01

Test	χ^2^	df	p
Overall model evaluation			
Likelihood ratio test	43.921	10	.001
Goodness-of-fit test			
Hosmer & Lemeshow	6.767	8	.562

*Note: N* = 276; Cox and Snell *R*^*2*^ = .147, Nagelkerke *R*^*2*^ (Max rescaled *R*^*2*^) = .197.

*∗p* < .05 after alpha Bonferroni correction.

### 3.6 Hypothesis 2: value priorities form distinguishable latent value profiles

**Figure 2:**
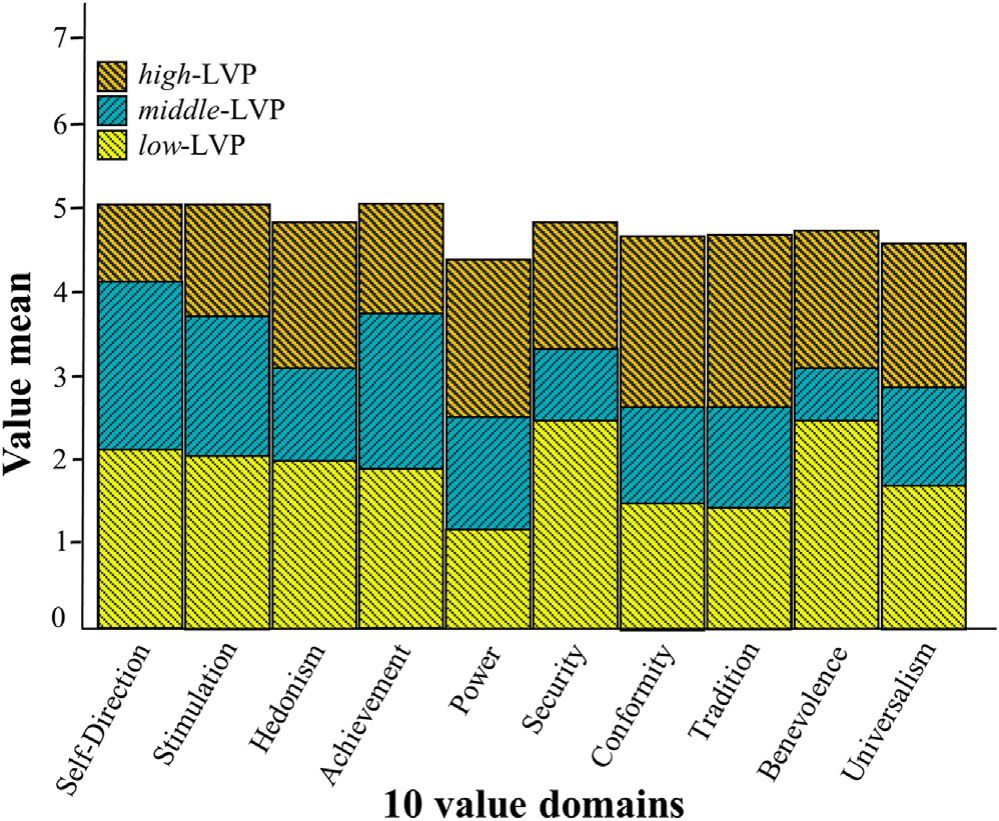
Patterns of means of the 10 value domains of the three value profiles participants associated with their meaningful experiences.

Using the poLCA package in RStudio (Linzer and Lewis, 2011) we conducted LPA to identify unobserved subgroups of participants differentiated by systematically diverging patterns of value ratings. We fitted a 1- up until 4-profile models and consulted their fit indices to decide on the number of profiles that best represented the data. Multiple fit indices dropped substantially with an additional number of profiles and started to increase in the 4-profiles model, indicating that the 3-profile solution was the best (Oberski, 2016) (Table 5). The rating response probabilities in each of the three profiles suggested three distinguishable latent value profiles that facilitated a conceptually meaningful interpretation. Accordingly, we interpreted that participants’ value ratings in relation to their meaningful experiences indicated their level of value orientation (LVO) or the degree to which the 10 universal values were important to them. In LVP-1, participants had *low*-LVO, as indicated by their value ratings (1); in LVP-2 participants had a *moderate*-LVO, as indicated by their value ratings (2–4); and in LVP-3 participants had *high*-LVO, as indicated by their value ratings (5–6) (Table 6). The probability of membership showed that 41% (*n* = 115, age *M* = 38.37, *SD* = 11.09, females = 64) were expected to belong to the *low*-LVO group, 38% (*n* = 105, age *M* = 37.25, *SD* = 12.16, females = 53) to the *moderate*-LVO group and 21% (*n* = 56, age *M* = 32.86, *SD* = 8.17, females = 21) to the *high*-LVO group. Figure 2 illustrates the patterns formed by the value means of the three LVO groups. These results supported our *Hypothesis 2*.

**Table 5:** Fit statistics of Latent Profile Analysis based on ratings of the 10 universal value domains (Schwartz, 1992) associated with meaningful experiences.

K	LL	AIC	BIC	SABIC	AWE	Entropy
1	-3911	7955	7935	7872	8105	1
2	-3426	7058	7027	6928	7292	.93
3	-3292	6863	6821	6688	7181	.89
4	-3280	6911	6858	6690	7314	.81

*Note. N* = 276; K = number of profiles; LL = log-likelihood; BIC = Bayesian Information Criterion; SABIC = Sample-size adjusted BIC; CAIC = Consistent Akaike Information Criterion; AWE = Approximate Weight of Evidence Criterion; BLRT = bootstrapped likelihood ratio test; *p* = *p*-value.

**Table 6:** Item-response probabilities in percentages for a 3-profile model, based on participants’ ratings of the 10 universal values.

Rating	1	2	3	4	5	6
**Profile 1**						

Self-Direction	**33.48**	29.11	13.67	8.57	10.07	5.10
Stimulation	**40.61**	26.71	17.14	6.08	4.16	5.29
Hedonism	**57.72**	16.01	6.79	9.77	4.29	5.42
Achievement	**56.21**	16.18	11.48	1.78	7.95	6.40
Power	**88.35**	9.22	00.00	0.00	1.54	0.89
Security	**37.00**	22.41	13.13	5.45	14.95	7.06
Conformity	**68.72**	14.47	13.23	3.59	0.00	0.00
Tradition	**76.64**	11.77	6.35	0.00	2.60	2.63
Benevolence	**42.26**	16.09	18.43	8.27	5.25	9.69
Universalism	**68.45**	10.97	7.63	8.49	1.81	2.64
**Profile 2**						

Self-Direction	1.92	16.23	31.07	**24.80**	19.13	6.86
Stimulation	3.73	12.13	31.12	**33.75**	19.27	0.00
Hedonism	16.83	19.92	**27.07**	19.41	10.37	6.40
Achievement	2.16	15.92	**30.67**	29.53	16.53	5.19
Power	21.60	**33.99**	19.11	18.29	6.04	0.95
Security	0.00	26.35	**37.55**	26.37	9.74	0.00
Conformity	14.19	**34.04**	24.52	21.34	5.92	0.00
Tradition	18.96	**28.95**	27.55	15.76	5.83	2.94
Benevolence	10.56	21.75	25.66	**30.33**	11.70	0.00
Universalism	1.77	29.29	**30.98**	20.29	5.76	1.91
**Profile 3**						

Self-Direction	0.00	0.00	3.46	19.58	**42.38**	34.58
Stimulation	0.00	1.74	1.74	15.21	**43.37**	37.94
Hedonism	1.55	0.00	5.18	28.66	29.83	**34.78**
Achievement	1.62	0.00	6.74	19.17	25.36	**47.11**
Power	14.03	0.00	1.76	29.12	**34.39**	20.70
Security	0.00	1.76	6.64	21.13	**37.70**	32.76
Conformity	7.20	0.00	10.96	21.78	27.29	**32.78**
Tradition	8.95	5.81	0.00	18.15	**36.14**	30.94
Benevolence	1.72	0.00	10.79	20.54	**34.18**	32.78
Universalism	1.72	3.34	10.29	20.96	**46.44**	17.21

*Note.* Text in bold highlights the highest value ratings in each of the three latent value profiles.

### 3.7 Hypothesis 3: average levels of well-being vary across latent value profiles

**Figure 3:**
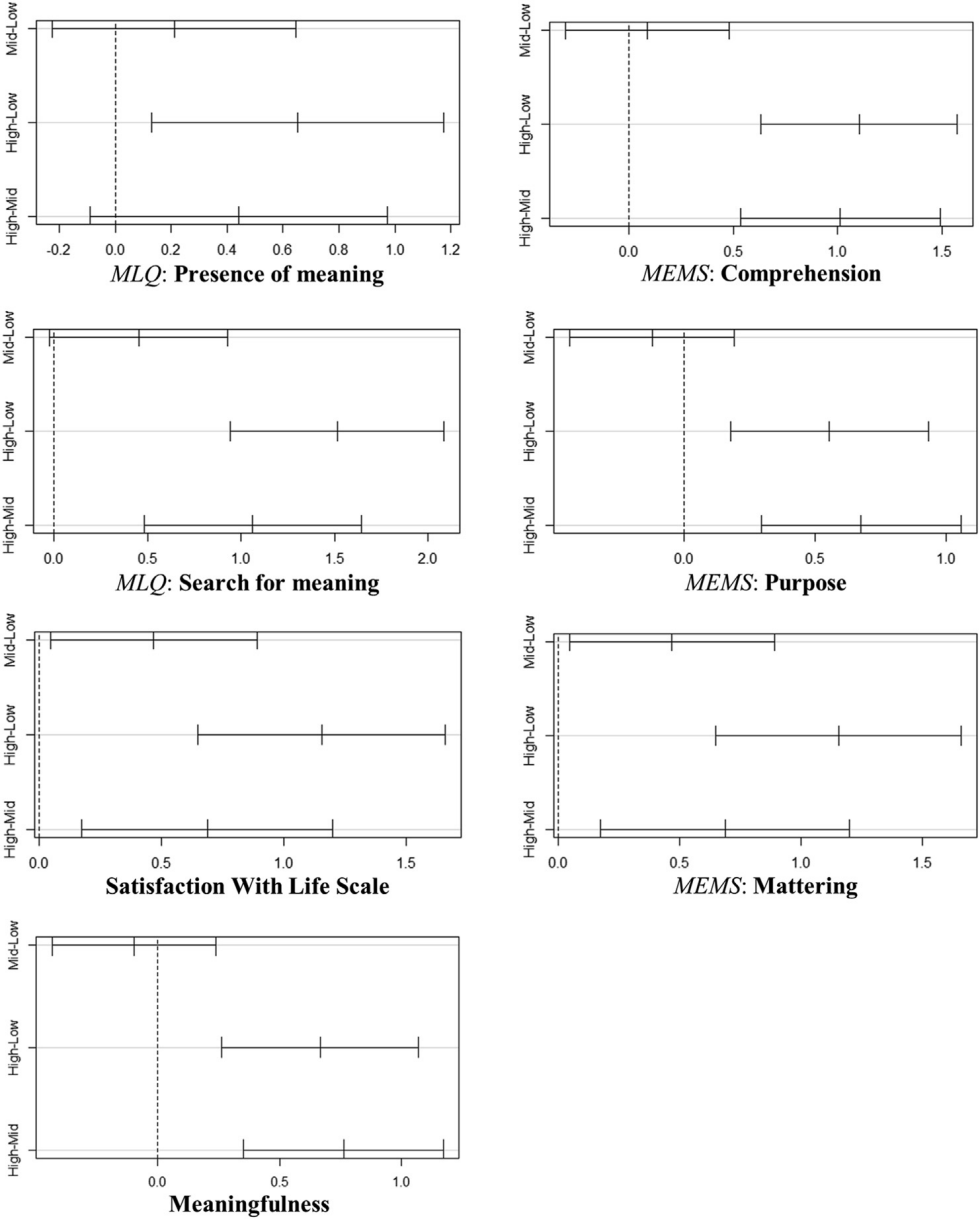
Multiple pairwise comparisons showing differences in the means of well-being measures across the three latent value profiles that participants associated with their meaningful experiences.

To investigate differences in the means of MLQ and MEMS subscales, the SWLS and in the ratings of *how meaningful* across the three LVO groups we conducted analysis of variance. The significant *F* values of the Levene's tests indicated that the homogeneity of variance assumption was violated. Hences, we conducted a series of Kruskal–Wallis tests (Kruskal and Wallis, 1952) to calculate the well-being measurements' means across groups. Table 7 reports the full results, which confirm our *hypothesis*. To identify where the differences between LVO groups occurred, we conducted nonparametric pairwise comparisons using the Dunn test (Dinno, 2015). Analysis revealed significant differences in the mean scores of the MLQ and MEMS subscales, the SWLS and ratings of “how meaningful” across the LVO groups. Differences emerged mainly between *high* to *moderate* and *high* to *low-*LVO groups (Figure 3).

**Table 7:** Kruskal-Wallis tests for differences between meaning and life satisfaction measures across the three latent value profile groups, denoting level of value orientation (LVO).

LVO	Mean/Standard Deviation					SWLS	Meaningful
	MLQ		MEMS				
	Presence	Search	Comprehension	Purpose	Mattering		
low	4.91/1.68	3.93/1.75	4.89/1.56	5.41/1.16	4.23/1.64	4.34/1.79	4.77/1.18
middle	5.12/1.13	4.39/1.40	4.98/1.00	5.28/0.92	4.71/1.16	4.70/1.17	4.68/1.08
high	5.56/1.01	5.45/1.04	6.01/0.78	5.96/0.66	5.38/0.77	5.66/1.10	5.44/0.65
Η(*df*) = χ^2^,	H(2) = 5.36,	H(2) = 33.99,	H(2) = 35.51,	H(2) = 19.76,	H(2) = 25,97,	H(2) = 30,27,	H(2) = 20.35,
*p*	<.014	<.001	<.001	<.001	<.001	<.001	<.001
*r*	.11	.33	34	.24	.28	.31	.25

*Note: N = 276; LVO =* level of value motivation; MLQ = Meaning in Life Questionnaire; MEMS = Multidimensional Existential Meaning Scale; SWLS = Satisfaction With Life Scale; χ^2 =^ Kruskal-Wallis chi-squared; *r* = effect.

## 4 Discussion

The present work contributes to previous research indicating that people share an intuitive understanding of meaning and that subjective judgments seem to be appropriate for capturing its phenomenological experience (King et al., 2006). In spite of suggestions that a definition is necessary when measuring meaning in life (e.g., De Vogler-Ebersole and Ebersole, 1985) as it is hard to define and verbalize (Huta, 2016; Steger et al., 2013), providing a definition of “meaningful” did not influence participants' responses to our measures. Moreover, a cross-sectional study supported three theory-driven hypotheses: First, that a source of meaning can be positively associated with one value while being negatively associated with another value. Second, that there are interindividual difference in participants' unobserved value patterns. Third, that people's latent value patterns can explain variance in well-being measures.

Converging with research indicating that people hold a common set of values (Schwartz, 1992) that contribute toward making sense of life (Reker and Wong, 1988), in the current study, 10 universal value domains were positively associated with the scales of MLQ (*cf.* Steger et al., 2006), MEMS and SWLS. Overall, values contributed positively to meaningful experiences, regardless of their underlying motivational orientation (*cf.* Reker and Wong, 1988; Schwartz, 1992). This robust relationship further emerged during the process of constructing a list of sources of meaning, where we identified that 52 items used in previous studies that investigated breadth of meaning constitute single values of the Schwartz's model (1992). Our findings did not converge with previous studies that report negative associations between certain values and the SWLS (e.g., the value of success that is under the domain of Achievement; Kasser and Ryan, 1996). Self-transcending values did not display a stronger association to meaning measures than conservation values did. Thus, depth of meaning was not associated with higher levels of meaning (*cf.* Reker and Wong, 1988) and values characterized mundane meaningful experiences regardless of their underlying motivational orientation. These results do not support research that characterizes certain values (e.g., Security) as “unhealthy” (*cf.* Sortheix and Schwartz, 2017). Regarding the breadth dimension of meaning, in line with previous research findings, which associate diversity and the number of sources of meaning with increased life satisfaction (Martela and Steger, 2016; Reker, and Wong, 1988; Wong, 2012), there were indications that widening the breadth of meaning may increase meaning and life satisfaction.

The current study replicated previous empirical evidence suggesting that “Family” is the most common source of meaning (e.g., Baum and Stewart, 1990; O'Connor and Chamberlain, 1996). In line with previous research suggesting that focusing on one value may decrease the importance of others (Bardi et al., 2009), “Family” was positively associated with one value domain (Tradition) and was negatively associated with another (Universalism). Participants who valued Tradition were likely to find meaning in family-related experiences whereas those who valued Universalism were not likely to find meaning in such experiences. Interestingly, in spite of its negative association to family-related experiences, Universalism correlated positively with the meaning and life satisfaction measures. This discrepancy may be explained by the dynamic relationship of values, as their importance fluctuates systematically (Bardi et al., 2009). Thus, when people place high importance on their immediate environment (values of Benevolence) they shift their focus away from matters concerning their broader social context (values of Universalism) (Besika et al., 2021). Similarly to findings indicating that a universal pairing of values to pleasurable experiences is unlikely to exist (Oishi et al., 1999), paired associations between sources of meaning and values cannot be generalized, as multiple values were associated to a single source of meaning. This observation converges with a recent theoretical development proposing that focusing on value priorities when investigating the relationship between values and well-being might constitute a limited approach to understanding the influence of the dynamic pattern of values on well-being (Besika et al., 2021).

In the present study, identifying unobserved value profiles and investigating their relationship to well-being measurements introduced an alternative approach to explaining interindividual differences in average levels of meaning and life satisfaction. Three distinct latent value profiles described the data and explained variance in well-being measurements across the three corresponding groups. The degree to which a cognitive pattern of 10 value domains (Schwartz, 1992) influenced daily meaningful experiences was associated with interindividual differences in the average levels people experienced meaning and life satisfaction. Participants with *high*-LVO (e.g., high level of value orientation) reported higher levels of meaning and life satisfaction compared to participants with *low*- and *moderate-*LVO. These results reflect previous findings suggesting that people's activities are meaningful when they align with their core values (McGregor and Little, 1998; Sheldon and Elliot, 1999) and converge with studies reporting that experiences congruent with personal values are satisfying (Oishi et al., 1999). Our findings also converge with another piece of evidence suggesting that when people's daily habits are close to their values people display strongly integrated self, high levels of increased self-esteem and a self-regulation style that tends to accomplish positive outcomes (Verplanken and Sui, 2019). Finally, the current results support recent findings indicating that people's value patterns, denoting the degree to which the 10 universal value domains influence their goals and actions consciously, influence their psychological balance and overall well-being (Besika et al., 2021).

The positive association of values to meaningful experiences and meaning measures supports the theoretical suggestion that meaning as a process has a dual movement. Moving downward, from the abstract level of cognitive functioning meaning constructs concrete experiences that generate a sense of satisfaction; moving upward, from the concrete level of behavior meaning deconstructs concrete experiences into abstract ideas, such as values (Mackenzie and Baumeister, 2014). This dual meaning movement implies an underlying mechanism that connects cognition to emotion and behavior.

### 4.1 Limitations

The present work indicates that people share an intuitive understanding of “meaningful”, adding validity to our results and to previous studies on meaning. However, we acknowledge a limitation in the design of the conditions that tested the effect of providing a definition of “meaningful” on subjective evaluations of meaning. To provide a clearer indication regarding the presence or absence of an effect of providing a definition, future studies may refrain from including any kind of definition in the control group. Furthermore, methodological limitations concern the susceptibility of participants' responses to retrospective biases that could have influenced participants’ ratings of the 10 value domains in relation to their manifestation in their experience over the previous two weeks. Longitudinal studies that involve samples of different cultures may further investigate the association of values to meaningful experiences. Daily diary reports may also facilitate investigations of fluctuations in the value patterns in response to daily challenges.

### 4.2 Conclusion

This article makes an incremental contribution toward understanding the important and yet underexplored relationship between values, meaning and life satisfaction. In line with previous studies that report meaning as a marker of well-being, our results show that a single source of meaning may be associated with multiple values. People find meaning mostly in family-related experiences and adding variety to the sources of meaning may increase life satisfaction and contribute toward value fulfillment. Moving beyond the typical focus on value priorities, the present work investigates people's latent value patterns, which provide new insights into the relationship between values and well-being. Three latent value profiles, denoting the level of people's value orientation, assessed by the level participants associated the 10 universal values with recent meaningful experiences, explained interindividual differences in the average levels of meaning and life satisfaction. In contrast to previous studies, our results indicate that values influence well-being as patterns and all 10 universal values may contribute positively to well-being, regardless of their underlying motivational orientation. In conclusion, focusing on value priorities may raise barriers in understanding the influence of values on daily experiences and overall well-being. This new perspective may stimulate future research as it implies an underlying mechanism that facilitates the systematic behavior of values and translates them into meaningful and satisfying experiences.

## Declarations

### Author contribution statement

Anastasia Besika: Conceived and designed the experiments; Performed the experiments; Analyzed and interpreted the data; Wrote the paper.

Jonathan W. Schooler: Conceived and designed the experiments; Wrote the paper.

Bas Verplanken: Conceived and designed the experiments; Wrote the paper.

Alissa J. Mrazek: Conceived and designed the experiments; Performed the experiments; Wrote the paper.

Elliott D. Ihm: Conceived and designed the experiments; Performed the experiments; Wrote the paper.

### Funding statement

This research was supported by the Fetzer Franklin Fund, awarded to Jonathan W. Schooler, with grant number 44069-59380.

### Data availability statement

Data will be made available on request.

### Declaration of interests statement

The authors declare no conflict of interest.

### Additional information

No additional information is available for this paper.

## Appendix A Supplementary data

Appendix_A **Appendix A:** a) Tables A1 – A3 that explain the process of formulating the list of sources of meaning and b) Tables A4 – A5 showing the 57 single values represented by the 10 value domains and the value descriptions we used in our study. Appendix_A
