# Supplementary material for: A relationship that makes life worth-living: levels of value orientation explain differences in meaning and life satisfaction
Source: Heliyon. 2022 Jan 24;8(1):e08802. doi: 10.1016/j.heliyon.2022.e08802 (PMC8802095; doi:10.1016/j.heliyon.2022.e08802)
Supplement: Appendix_A — Appendix A: a) Tables A1 – A3 that explain the process of formulating the list of sources of meaning and b) Tables A4 – A5 showing the 57 single values represented by the 10 value domains and the value descriptions we used in our study. [file mmc1.pdf]

APPENDIX A

A comprehensive list of sources of meaning and value descriptions.

Table A1. Sources of meaning: Reviewed previous studies on sources of meaning, since 1980.

Publication	Sources of meaning	Sample
Bar-Tur et al. (2001	Family and Communal Values / Materialistic Concerns /Autonomy and independence / Interpersonal relationships / Communal activities /Attainment of tranquility /Self-development and leisure / Family relationships / Leisure activities with others / Being with animals / Good relationhips with spouce	Young to old adults (Jews and Arabs)
Baum & Stewart (1990)	Work / Love and Marriage / Births of Children / Independent Pursuits / Accidents, Illnesses - Death / Separations-Divorces / Major Purchases	Young and older adults
Debats (1999)	Relationshisp / Family, Partner / Lover / Friends /Combinations (of relationships) / Lifework / Personal well-being (appreciation of life) / Health / Self-actualisation, (tangible and intangible goals / talents) / Service, Beliefs (religious / spiritual, socila/ political) / Materiality / Future - hope	Young adults
DeVogler & Ebersole (1980)	Relationships / Service / Growth / Belief / Existential-Hedonistic / Obtaining / Expression / Understanding	College students
DeVogler & Ebersole (1983)	Relationships / Activities / Health / Obtaning / School / Appearance / Belief / Growth / Service / Pleasure / Miscellaneous	Adolescents
Ebersole & DeVogler (1981)	Relationships / Belief / Health / Growth / Life work / Service / Understanding / Pleasure / Expression / Obtaining / Miscellaneous	Adults
Ebersole & Depaola (1987)	Relationships / Service / Belief / Obtaining / Growth / Health / Life Work / Pleasure / Appearance /Activities / School / Miscellaneous	Adolesents
Fegg et al. (2008)	Family / Work / Studying / Friends / Partner / Leisure time / Religiosity / Health / Animals / Nature / Finances / Well-being / Hedonism / Altruism / Home (garden)	University students
Lambert et al. (2010)	Happinessa / Friends / Family / Religious faith / Achievements / Self-acceptance / Personal growth / Self-worth / Justice-fairness / Personal goals / Intimacy / Helping others	Undergraduate students
Reker (1996)	Engaging is personal relationships with family and friends, Feeling financially secure, Preserving human values and ideals, Meeting basic everyday needs, Participation in leisure activities / Interest in human rights (humanistic concerns) / Being of service to others / Leaving a legacy for the next generation / Preserving cultrue and tradition / Taking part in creative activities / Experiencing personal growth / Participating in hedonistic activities / Interest in social causes / Being acknowledged for personal achievements / Acquiring material possessions in order to enjoy the good life / Taking part in religious activitites	Older adults
Reker & Guppy (1988)	Personal relationships / Altruism / Religious activities / Creative activities / Personal growth / Metting basic needs / Pleasurable-leisure activities / Personal achievement / Legacy / Enduring values or ideals / Traditions and culture / Social-political causes	Adults
O'Connor & Chamberlain (1996)	Relationship with People / Creativity / Religious-Spiritual / Personal Development / Social-Political / Relationships with Nature	Mid-life adults
Schnell (2009)	Self-transcendence (*Vertical:* Explicit religiosity / Spirituality - *Horizontal:* Social commitment / Unison with nature / Self-knowledge / health / generativity) / Self-actualization (Challenge / Individualism / Power / Development / Achievement / Freedom / Knowledge / Creativity) Order (Tradition / Practicality / Morality / Reason) / Well-being / Relatedness (Community / Fun / Love / Comfort / Care / Attentiveness / Harmony)	Adolescents and older adults (German)
Steger et al. (2013)	Hobby - Leisure / Relationships / Pets / Possessions / Everyday necessities / Religion / Education / Technology / Organization-Activities / Physical environemnt / Occupation - work / Nature / Values / Future aspirations / Self-love, Love / Trust / Heritage / Honesty / Family traditions / Memories	Undergraduate students
Unknown study	Supporting my children / Helping my mum / Love / Giving food to homeless people / Swimming / My career / Trust / My child growing up / Gardening / Walking / Going to church / My partner.	Convenient sample

Note: List includes only studies that used unique sources of meaning

References

Bar-Tur, L., Savaya, R., & Prager, E. (2001). Sources of meaning in life for young and old Israeli Jews and Arabs. *Journal of Aging Studies, 15* (3), 253-269.

Baum, S. K., & Stewart Jr, R. B. (1990). Sources of meaning through the lifespan. *Psychological Reports, 67* (1), 3-14.

Debats, D. L. (1999). Sources of meaning: An investigation of significant commitments in life. *Journal of Humanistic Psychology, 39* (4), 30-57.

Devogler, K. L., & Ebersole, P. (1980). Categorization of college students' meaning of life. *Psychological Reports, 46* (2), 387-390.

Devogler, K. L., & Ebersole, P. (1983). Young adolescents' meaning in life. *Psychological Reports, 52* (2), 427-431.

Ebersole, P., & Devogler, K. L. (1981). Meaning in life: Category self-ratings. *The Journal of Psychology, 107* (2), 289-293.

Ebersole, P., & Depaola, S. (1987). Meaning in life categories of later life couples. *The Journal of Psychology* , 121(2), 185-191.

Fegg, M. J., Kramer, M., L'hoste, S., & Borasio, G. D. (2008). The Schedule for Meaning in Life Evaluation (SMiLE): Validation of a new instrument for meaning-in-life research. *Journal of Pain and Symptom Management* , 35(4), 356-364.

Lambert, N. M., Stillman, T. F., Baumeister, R. F., Fincham, F. D., Hicks, J. A., & Graham, S. M. (2010). Family as a salient source of meaning in young adulthood. *The Journal of Positive Psychology* , 5(5), 367-376.

O'Connor, K., & Chamberlain, K. (1996). Dimensions of life meaning: A qualitative investigation at mid-life. *British Journal of Psychology, 87* (3), 461-477.

Reker, G. T. (1996). Sources of Meaning Profile- Revised.

Reker, G. T., & Guppy, B. (1988). Sources of personal meaning among young, middle-aged, and older adults. In *Annual Meeting of the Canadian Association on Gerontology.* Halifax, Nova Scotia.

Schnell, T. (2009). The Sources of Meaning and Meaning in Life Questionnaire (SoMe): Relations to demographics and well-being. *The Journal of Positive Psychology, 4* (6), 483-499.

Steger, M. F., Shim, Y., Rush, B. R., Brueske, L. A., Shin, J. Y., & Merriman, L. A. (2013). The mind's eye: A photographic method for understanding meaning in people's lives. *The Journal of Positive Psychology, 8* (6), 530-542.
Unknown study, 2016: The study was conducted by students.

Table A2. Sources of meaning: Generated a total of 106 sources of meaning from the literature, including the 12 items from our study.

1. Accidents - Illnesses[a]
2. Achievement[a]
3. Animals[a] / Pets[c]
4. Appearance[a]
5. Appreciation[c]
6. Awe[a]
7. Birth[a]
8. Care[a]
9. Challenge[b]
10. Community[a]
11. Connection[b]
12. Creativity[b]
13. Death[a]
14. Education[a] / Knowledge[c] / School[c] / Studying[c]
15. Fair treatment[b] / Justice[b] / Fairness[b]
16. Faith[b] / Religion[b] / Belief[b] / Going to Church[b]
17. Family[a] / Bringing up children[c]
18. Flow[a]
19. Freedom[b] / Autonomy[b]
20. Friends[a]
21. Generativity[b]
22. Gratitude[b]
23. Hedonistic activities[a] / Leisure[b] / Fun[b] / Indulgence[b]
24. Helping others[a] / Service[b] / Altruism[b] / Generosity[c] / Humanistic concerns[b] / Food to homeless[b]
25. Hobbies[a] / Activities[c] / Swimming[c]
26. Home[a]
27. Inspiration[b]
28. Legacy[b]
29. Life changing[b]
30. Life satisfaction[b]
31. Love[b]
32. Marriage[a]
33. Mindfulness[a]
34. Money[a] / Finances[b]
35. Nature[a] / Gardening[c]
36. Necessities[a] / Comfort[c]
37. Novelty[b]
38. Partner[a]
39. Peace[b] / Safety[b] / Harmony[b]
40. Personal goals[b] / Purpose[b] / Independent Pursuits[b] / Future aspirations[b]
41. Personal growth[b] / Reaching personal potential[b] / Self[b]
42. Physical Environment[a]
43. Pleasure[b] / Happiness[b] / Feel good[b]
44. Possessions[a] / Major Purchases[c] / Obtaining[c]
45. Power[b]
46. Quality time[a]
47. Reflection[a]
48. Relationships[a] / Restoring a relationship[c]
49. Responsibility[b]
50. Savoring[a]
51. Self-awareness[b] / Self-acceptance[b]
52. Self-care[b]
53. Self-worth[b]
54. Separation[a] / Divorce[c]
55. Societal-political[a]
56. Spirituality[b] / Self-transcendence[b]
57. Support[b] / Helping members of the family (child, parent)[c]
58. Synchronicity[a]
59. Technology[a]
60. Tradition[b]
61. Validation[b]
62. Well-being[a] / Health[b]
63. Work[a] / Occupation[c] / Career[c]

Note: [a] = semantically unique item, [b] = items similar to single values, [c] = items not semantically unique.

Table A3. Step 3: Definitions of the semantically unique sources of meaning (35).

1. **Accidents – Illnesses**: experiencing an unexpected and undesirable event that resulted in poor health, harm, injury, damage or loss.
2. **Animals**: caring for an animal that you keep as a companion or come to contact with.
3. **Appearance**: being concerned with the outward impression of yourself or others.
4. **Awe**: feeling amazed or being overwhelmed with wonder.
5. **Birth**: experiencing the birth of a child in your family or otherwise.
6. **Care**: giving your full attention to someone or something else than yourself.
7. **Community**: being involved in a group that shares common characteristics or interests.
8. **Death**: losing someone important or someone you were close to.
9. **Education**: becoming familiar with facts, truths or principles through study or investigation.
10. **Family**: people you are related to or feel close to, like parents or children whether you live together or not.

11. **Flow**: losing sense of time while doing something smoothly and effortlessly.
12. **Friends:** people you are emotionally attached to or are associated with.
13. **Hedonistic activities**: things that you find intensely pleasurable
14. **Helping others:** being kind and generous to others, having selfless concerns for others.
15. **Hobbies**: activities you do in your free time
16. **Home:** the place in which you live or your affections are centred.
17. **Marriage:** being legally bound to your partner and having a family with them.
18. **Mindfulness:** focusing your full attention in the present moment experiencing thoughts, feelings and sensations without judging
19. **Money**: experiencing financial comfort or lack of.
20. **Nature:** enjoying the elements of the natural world or feeling connected to them.
21. **Necessities**: having everything that is needed for your survival and basic needs.
22. **Partner:** someone you share a romantic relationship with or have a common purspose.
23. **Personal goals**: dreaming about the future and making plans.
24. **Physical Environment:** a location that you occupy, visit or use for a specific purpose.
25. **Possessions**: acquiring material objects, making major purchases.
26. **Quality time:** spending time with others or self, giving your undivided attention.
27. **Reflection**: being able to reflect on your experiences while knowing your character, feelings, motives and desires.
28. **Relationships**: experiences involving people you feel connected to and have a special bond with.
29. **Savoring**: experiencing things with all your full attension
30. **Separation**: the ending or interruption of a relationship.
31. **Societal-political:** things that relate to the wider society to the government or public affairs.
32. **Synchronicity**: experiencing a coincidence of events.
33. **Technology:** using a computer, phone etc. as tools of everyday life
34. **Well-being**: experiencing a good state of being with soundness of body and mind.
35. **Work**: exercising your skills and knowledge as a means of earning a living.

Table A4. The 10 basic value domains (Schwartz, 1992; 2012) and the 57 individual values contained in each domain.

1. **Self-Direction**: Creativity / curious / freedom / choosing own goals / independent / private life
2. **Stimulation** : Daring / a varied life / an exciting life
3. **Hedonism**: Pleasure / enjoying life / self-indulgent
4. **Achievement**: Successful / capable / ambitious / influential / intelligent / self-respect
5. **Power**: Social power / authority / wealth / preserving my public image / social recognition
6. **Security**: Clean / national security / social order / family security / reciprocation of favors / healthy / sense of belonging
7. **Conformity**: Politeness / honoring parents and elders / obedient / self-discipline
8. **Tradition**: Devout / accepting portion in life / humble / moderate / respect for tradition
9. **Benevolence**: Helpful / honest / forgiving / loyal / responsible / true friendship / a spiritual life / mature love / meaning in life
10. **Universalism**: Protecting the environment / a world of beauty / unity with nature/ broad-minded / social justice / wisdom / equality / a world at peace / inner harmony

Table A5. Descriptions of the 10 basic values based on the single values they represent.

**Self-Direction**:	Experiences that enhanced your independent thought and action and contained elements such as: creativity, freedom (autonomy), purpose, personal growth, responsibility, self-acceptance, self-awareness, elf-care, self-worth and validation.
**Stimulation**:	Experiences that gave you a sense of excitement and contained elements such as: novelty, challenge, inspiration, life changing and self-transcendence.
**Hedonism**:	Experiences that gave you a sense of sensuous gratification and contained elements such as: happiness, fun, leisure, pleasure, feel good, indulgence and life satisfaction.
**Achievement:**	Experiences that gave you a sense of personal success and contained elements of: reaching personal potential and legacy.
**Power**:	Experiences that enhanced your social status and prestige and gave you a sense of power, control and dominance over people or resources.
**Security**:	Experiences that enhanced (or threatened) your sense of safety and stability and contained elements such as: connection, comfort, intimacy, harmony, understanding, self, well-being.
**Conformity**:	Experiences that restraint your actions, inclinations, and impulses like**:** obedient, self-discipline, politeness, honoring parents and elders.
**Tradition**:	Experiences that enhance your sense of respect, commitment, and acceptance of the customs and ideas of your culture and contain elements such as religion and belief.
**Benevolence**:	Experiences that preserve and enhance the welfare of those you are in touch with and contain the elements of: altruism, appreciation, generosity, gratitude, love, service and support.
**Universalism**:	Experiences that enhance your understanding, appreciation, tolerance, and protection of the welfare of *others* and of nature and contain the elements such as: justice, fairness, humanistic concern, peace and spirituality.
