# Supplementary material for: A relationship that makes life worth-living: levels of value orientation explain differences in meaning and life satisfaction
Source: Heliyon. 2022 Jan 24;8(1):e08802. doi: 10.1016/j.heliyon.2022.e08802 (PMC8802095; doi:10.1016/j.heliyon.2022.e08802)
Supplement: Appendix_B — Appendix B: Table 1. [file mmc2.pdf]

APPENDIX B

Table 1.
Kruskal-Wallis tests tested differences between meaning and life satisfaction measures across depth of meaning or the four motivational orientations that underlie the 10 value domains.

	Mean / Standard Deviation					
	MLQ		MEMS			SWLS
MOV	*Presence*	*Search*	*Compreh.*	*Purpose*	*Mattering*	
Conservation	5.11 / 1.38	4.43 / 1.58	5.14 / 1.31	5.47 / 1.01	4.65 / 1.37	4.75 / 1.52
Self-enhancement	5.12 / 1.38	4.43 / 158	5.14 / 1.30	5.47 / 1.01	4.65 / 1.37	4.75 / 1.52
Openness to change	5.12 / 1.38	4.43 / 1.58	5.14 / 1.30	5.47 / 1.01	4.65 / 1.37	4.75 / 1.52
Self-transcendence	5.12 / 1.38	4.43 / 1.58	5.14 / 1.30	5.47 / 1.01	4.65 / 1.37	4.75 / 1.52
H(*df*) = χ^2, *p*	H(3)= .00, 1	H(3) =.00, 1	H(3) =.00, 1	H(3) =.00, 1	H(3) =.00, 1	H(3) =.00, 1

Note: N = 276; MOV = Motivational orientation of values; MLQ = Meaning in Life Questionnaire; MEMS = Multidimensional Existential Meaning Scale; SWLS = Satisfaction With Life Scale; H = test statistic for Kruskal-Wallis test; *df* = degrees of freedom; χ^2 = Kruskal-Wallis chi-squared.
